# The Beat

**Published:** 2011-02

**Authors:** Erin E. Dooley

## NY State Tanning Bed Regs

Newly implemented regulations on indoor tanning salons put New York State in the company of almost a dozen states by restricting indoor tanning for children.^1^ New York children under age 14 may no longer use such facilities, and older teenagers must have signed parental consent. Adults must acknowledge they are aware of the hazards of indoor tanning and receive instruction in the use of tanning devices. Among other requirements, tanning facility operators in New York State must provide free protective eyewear to customers and ensure customers use it.

**Figure:**
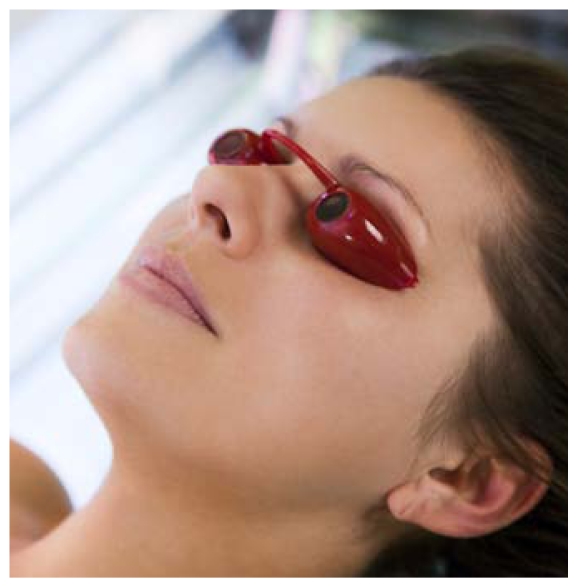
Protective eyewear is now required for New York tanning-bed users.

## Air Filtration Devices for Port Community Schools

California’s South Coast Air Quality Management District has approved the installation of high-performance air filtration devices at 47 schools in Wilmington, a community heavily polluted by shipping and transport activity at the Port of Los Angeles.^2^ The decision follows a demonstration project showing the devices removed up to 90% of diesel and ultrafine particles from air inside the classrooms. Funding for the project comes from a settlement with the City of Los Angeles and community groups to mitigate environmental impacts of the TraPac Container Terminal Expansion Project at the Port of Los Angeles. The devices will be installed this spring.

**Figure:**
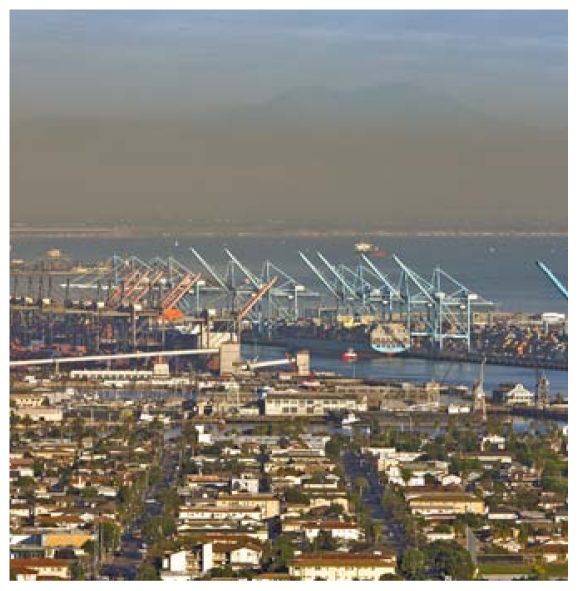
Los Angeles Harbor, near Wilmington.

## Tap Water and Hypospadias

Inconclusive evidence to date has suggested a potential link between exposure to trihalomethanes (THMs), a tap water disinfection by-product, and hypospadias, a genital birth defect that affects 70 in 10,000 male births. A study of 471 mother–son pairs showed maternal exposure to THMs did not explain the risk of hypospadias but that women who drank more than a liter of cold tap water per day had a 70% higher chance of having a baby with the birth defect, a finding the authors say needs further exploration.^3^ They also stress the importance of adequate fluid intake for pregnant women.

## Funds to Study Combined Effects of Social Stressors, Pollutants

In January 2011 the EPA announced new grants in support of a nationwide interdisciplinary effort to study poor and underserved communities that have extensive pollution problems.^4^ Funded projects will examine social and societal factors that may modify how pollutants affect human health. The program will focus on multiple pollutants, where programs in the past have generally focused on single chemicals.

**Figure:**
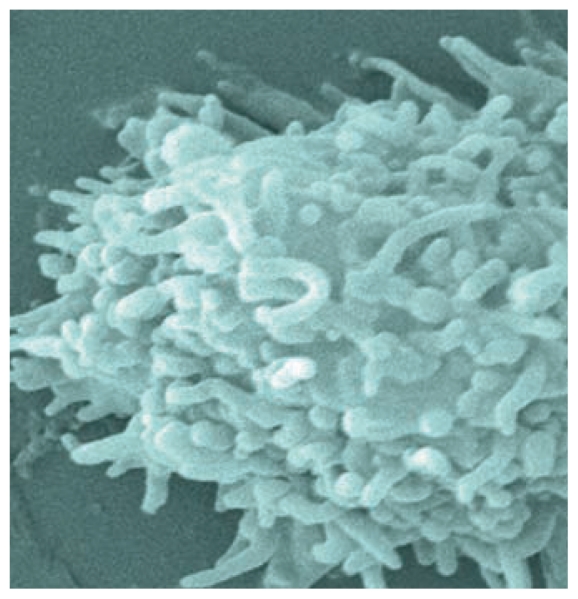
*Acanthamoeba* is a relatively common FLA.

## Free-Living Amoebas Common in Water Systems

A growing body of research has shown so-called free-living amoebas (FLAs) increase the quantity and virulence of water-based pathogens such as *Legionella* and *Mycobacterium* spp. A recently published review shows FLAs can break through water treatment barriers and enter drinking water distribution systems, where they can colonize and grow, especially in reservoirs and home plumbing.^5^ FLAs were found in 45% of the water samples in the studies reviewed, reflecting treated drinking water systems from around the world. The health impact of these prevalent FLAs has yet to be determined.
